# Ultrasound diagnosis of appendiceal tumor with synchronous liver metastasis mimicking acute appendicitis and liver abscess

**DOI:** 10.1007/s40477-025-01021-y

**Published:** 2025-04-29

**Authors:** L. Tarantino, P. Di Sario, R. A. Nasto, A. Nasto, L. Pellegrini, S. Sellitto

**Affiliations:** 1 Department of Surgery, Interventional Hepatology Unit, Luigi Curto Hospital, Polla, Italy; 2 Department of Medicine, Institute of Radiology, University of Udine, Udine, Italy

**Keywords:** Appendiceal tumor, C.E.U.S., Ultrasound, CT, Liver metastasis, Colorectal cancer, Appendiceal neoplasms, Mucinous neoplasia, Incidental neoplasms

## Abstract

Appendiceal tumors (AT) are rare gastrointestinal cancers with high incidence of synchronous and metachronous colorectal cancer metastases. AT are rarely associated with hepatic metastases. Sometimes, metastases are the first evidence of the tumor. We report the clinical, surgical and imaging records of a female patient, 81 years old, admitted for a suspect liver abscess, only subsequently diagnosed as metastasis from an undiagnosed AT. Ultrasound (US) based imaging techniques, such as Color-Doppler Ultrasound, Contrast enhanced US, US-guided biopsy of the hepatic nodule played an important role in the assessment of the definitive diagnosis.

## Backgound

Appendiceal tumors (AT) are rare gastrointestinal cancers. Their incidence ranges between 0.12 and 1.3 per one million population and represent only the 0.5% of all gastrointestinal neoplasms [1, 2]. The 65% of AT are of neuroendocrine origin, while adenocarcinomas constitute approximately 20% of these tumors. Distant metastases can present as synchronous or metachronous and sometimes involve the liver parenchyma. Liver metastases can represent the first evidence of the tumor and only subsequent imaging studies detect the primitive location. We report the clinical, surgical and imaging records of a patient with a suspect liver abscess, only subsequently diagnosed as metastasis from an undiagnosed AT. In particular, we stress the important role played from ultrasound (US)-based imaging techniques in the diagnostic pathway to the definitive diagnosis [3, 4].

## Case report

An 81-year-old female patient, with anamnesis of hypertension and heart disease, presents recent onset of fever, abdominal pain, weakness, constipation and weight loss was admitted at our Institution for suspected liver abscess. Blood test showed leukocytosis, high CRP, increased AST, ALT and bilirubin. A US examination performed in another Institution showed a large Inhomogeneous collection in the right hepatic lobe, with internal septa suggesting liver abscess.

**Fig. 1:**
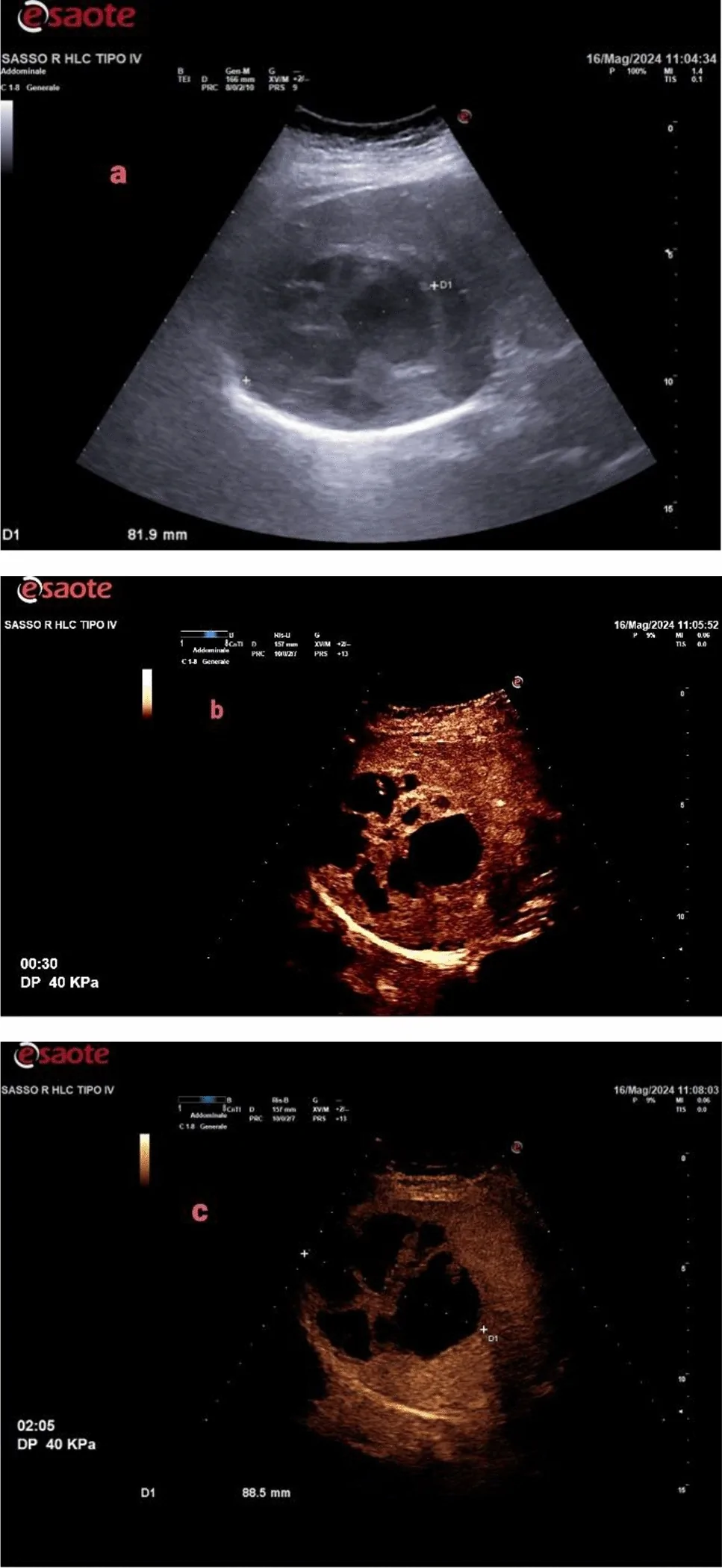
US (**a**), CEUS arterial phase (**b**), CEUS late phase (**c**) of the liver lesion

**Fig. 2:**
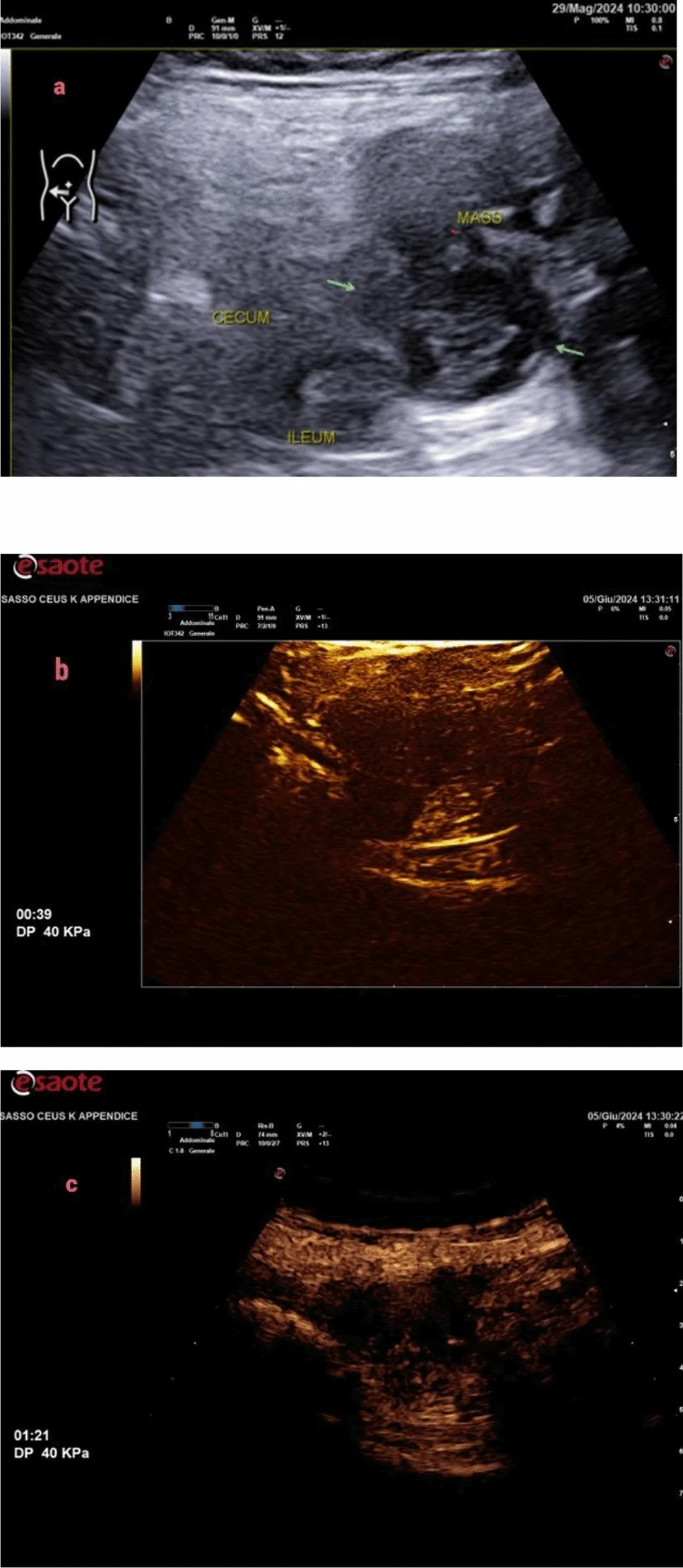
US (**a**), CEUS arterial phase (**b**), CEUS late phase (**c**) of the appendiceal lesion

**Fig. 3:**
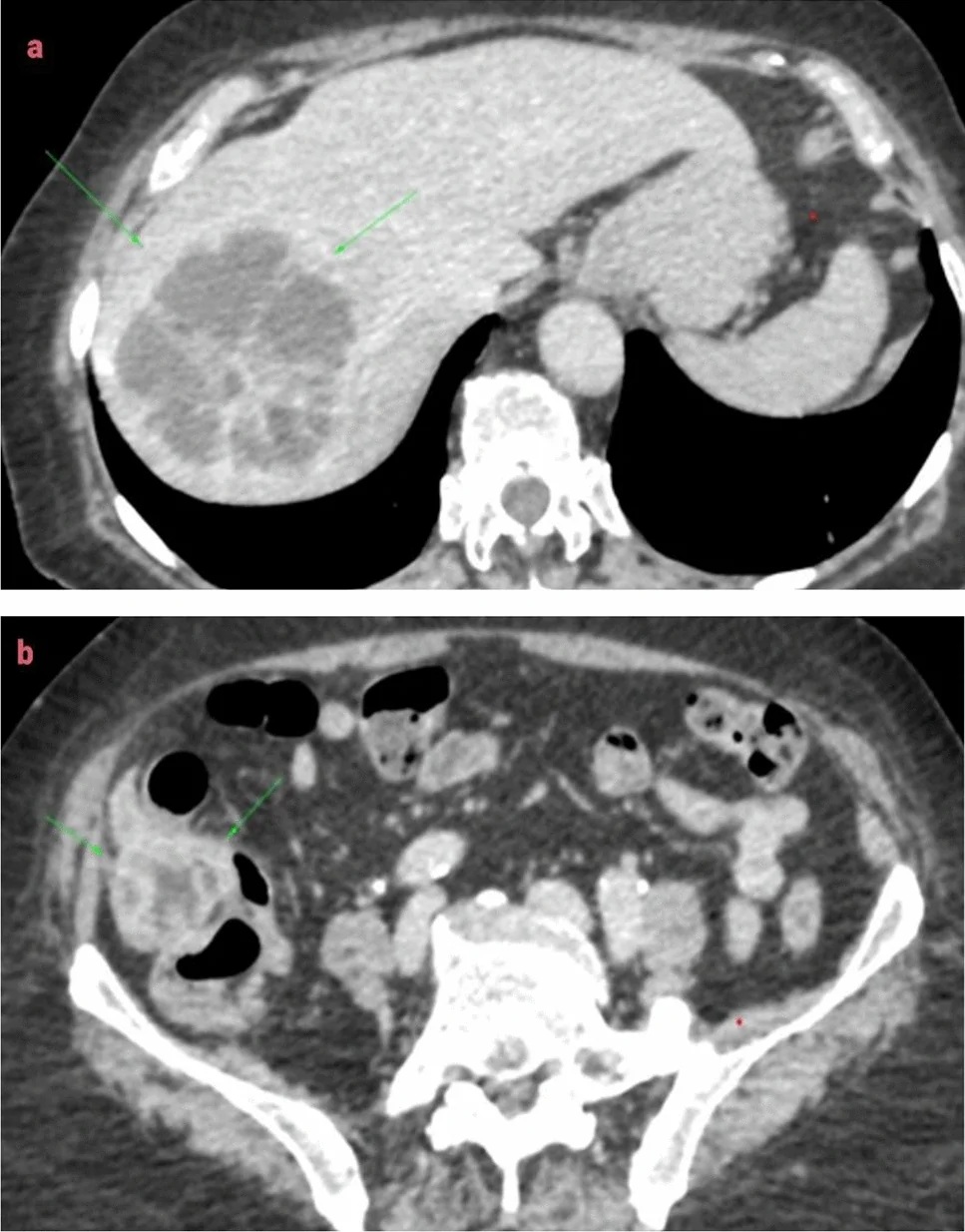
CT image of the liver (**a**) and appendiceal lesion (**b**)

**Fig. 4:**
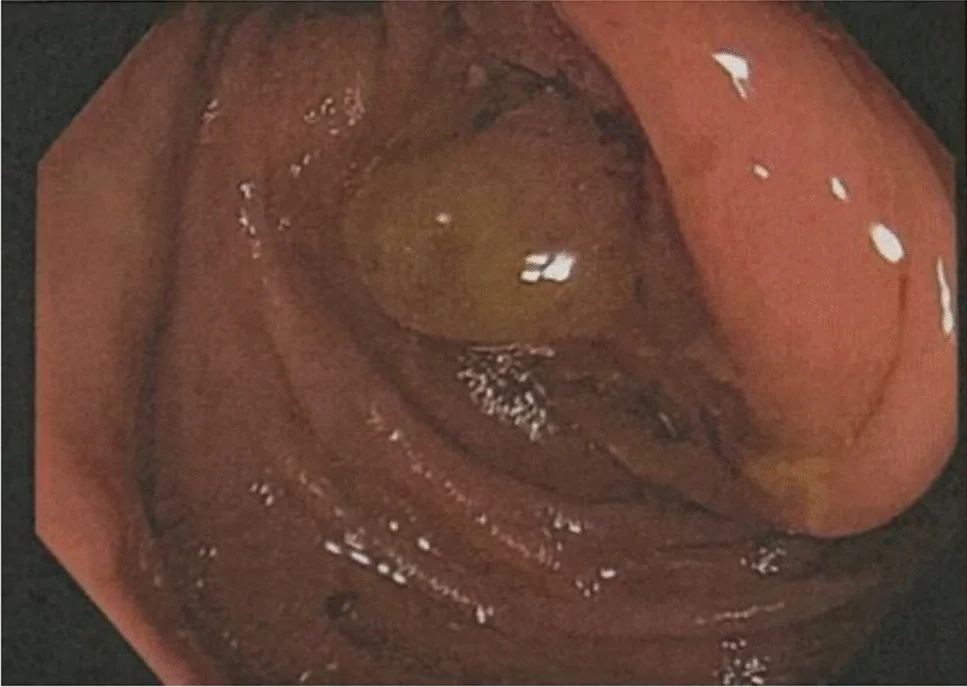
Colonoscopic appearance of the appendiceal lesion

**Fig. 5:**
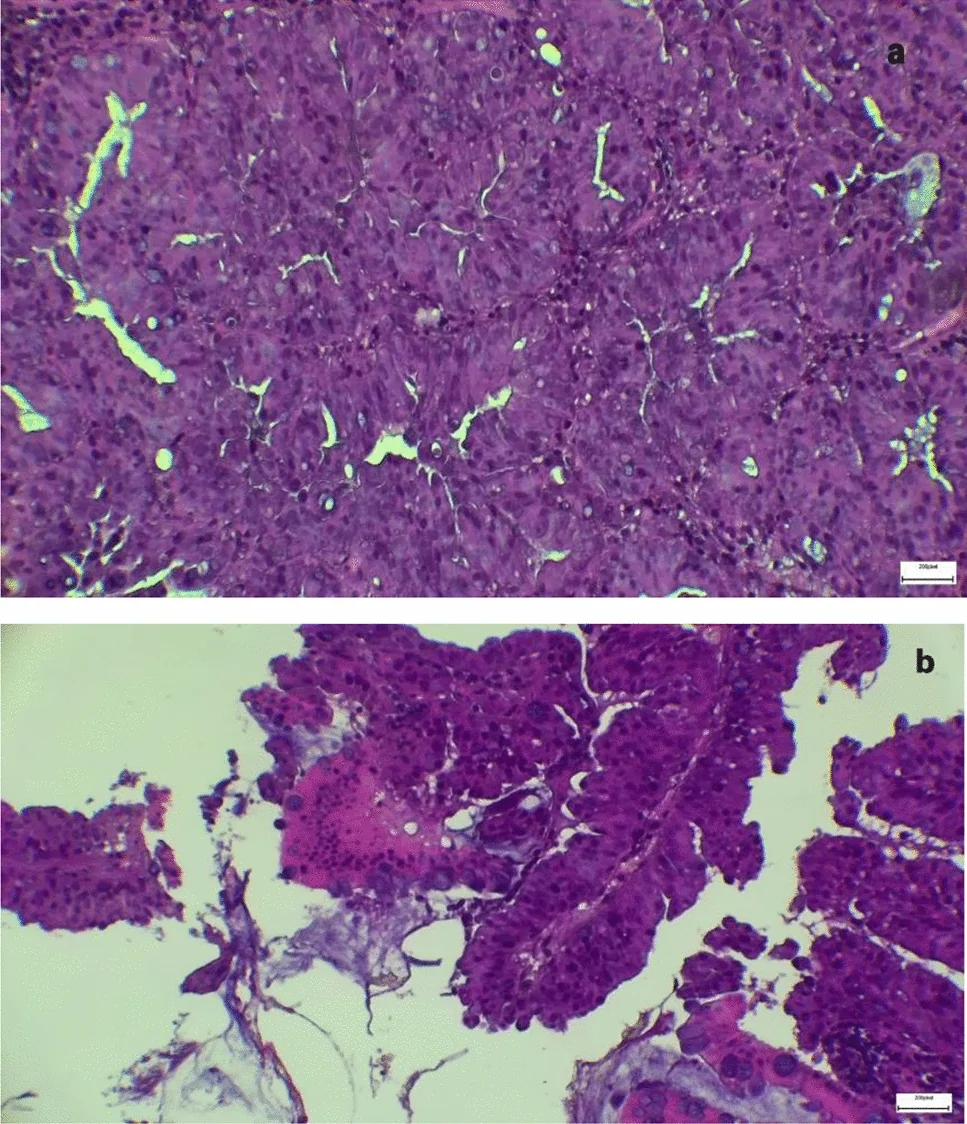
Liver (**a**) and appendiceal (**b**) biopsy microscopic images

On the day of admission, the patient underwent US with a 3.5 MHz convex probe (MyLab™X9 ESAOTE, Italy) that described a large complex mass in the right hepatic lobe, apparently capsulated, with multiple prevalent anechoic "fluid-filled-like" areas, separated from thick septa with solid roots on the capsule and vascularized at color-doppler study. These features suggested a possible abscess in a liver tumor mass. In the same session, we performed contrast enhanced US (CEUS) (Sonovue, Bracco, Milan Italy) that showed an early, intense enhancement of the septa in the arterial phase, venous and late phase wash-out, consistent with tumor neoangiogenesis in an evolutive rather than necrotic lesion [5] (Fig. 1a–c). The US and CEUS pattern suggested a possible metastasis from cystoadenocarcinoma. In suspicion of an ovarian neoplasm, US examination was extended to the pelvis, and an expansive mass in the right ileo-cecal region was detected. This second lesion presented with the same US and CEUS features of the liver mass (Fig. 2a–c). The intralesional hypo anechoic areas, apparently fluid, could be explained with the presence of mucinous material in a colonic adenocarcinoma [6, 7]. The US pattern and the strict adhesion to the cecum, suggested an exophytic mucinous adenocarcinoma of the cecum with synchronous hepatic metastasis. The patient underwent three phase enhanced CT scans that confirmed a large, partly colliquate, hepatic mass in the VII—VIII segment, and an exophytic wall thickening with inhomogeneous contrast enhancement of the cecum (Fig. 3a, b) [8]. Colonoscopy showed a solid appendicular mass partly protruding in the cecum (Fig. 4) on which biopsies were performed. In order to confirm the metastatic nature of the hepatic lesion, a fine needle US guided biopsy of the hepatic mass was performed [9, 10]. The microscopic assessment of the colonic and liver specimens were both suggestive of mucinous neoplasm (Fig. 5a, b) and the definitive diagnosis of "Mucinous neoplasm of the appendix with hepatic metastasis" was made. The patient before evaluation the best procedure to adopt at multidisciplinary council, died for cardiovascular issues.

## Discussion

AT include a heterogeneous group of tumors with four major histopathologic subtypes: mucinous neoplasm (low-grade and high-grade appendiceal mucinous neoplasm), adenocarcinoma (mucinous and non-mucinous), goblet cell adenocarcinoma, and neuroendocrine neoplasm. Appendiceal mucinous neoplasms are present in 0.2–0.3% of appendectomy specimens and are frequently an incidental finding. AT usually occurs during the sixth decade of life, and mucinous tumors can clinically mimic acute appendicitis. They are relatively difficult to diagnose, especially when neoplasms are limited to the appendix, therefore the diagnosis is frequently made incidentally following appendectomy for presumed appendicitis after histologic review of the resected specimen [11]. All types of appendiceal tumors have a high incidence of synchronous and metachronous colorectal cancer. In a retrospective study, including patients who underwent an appendectomy between December 2003 and December 2014, seventy-two mucinous tumors of the appendix were identified among 7.717 patients reviewed, resulting in a prevalence of 0.9%. An incidental diagnosis was made in 43% of patients. In another study, 22 (30.5%) patients showed a synchronous or metachronous colorectal cancer. Worth of note, an increased risk of ovarian mucinous tumors was identified in patients with a diagnosis of appendiceal mucinous neoplasm. Another possible progression of appendiceal mucinous AT is pseudomyxoma peritonei [11, 12]. Hepatic metastases from primary AT are rarely reported. Liver metastases can occur as the first presentation of the tumor. In such cases, the diagnosis of the primary neoplasm can be challenging. CT, MRI and PET/CT are indicated for this purpose. In our patient, presenting with fever and hepatic solid/fluid mass, the diagnosis of admission was a suspect liver abscess. However, US and CEUS studies promptly addressed the correct diagnosis of malignant focal liver lesion by detection of characteristic color-doppler and contrast enhancement patterns. Worth of note, the arterial enhancement of intralesional septa and their wash-out in the late phase, were particularly evident in the CEUS rather than in the CT study. The real-time nature of CEUS in comparison with the static appraisal of CT could explain the important value of CEUS in this case [13, 14]. Therefore, the suspicion of a mucinous neoplasm of the liver prompted the search for a possible ovarian or colonic tumor. US and CEUS were particularly effective in the detection of the primary tumor. However, the correct assessment of tumor origin was determined by colonoscopy guided biopsy. Moreover, US guided biopsy confirmed the secondary nature of the hepatic lesion.

## Abbreviation

AT: Appendiceal tumor
